# Supplementary material for: A scenario modeling pipeline for COVID-19 emergency planning
Source: Sci Rep. 2021 Apr 6;11:7534. doi: 10.1038/s41598-021-86811-0 (PMC8024322; doi:10.1038/s41598-021-86811-0)
Supplement: Supplementary file 1 — Supplementary Information. [file 41598_2021_86811_MOESM1_ESM.pdf]

A scenario modeling pipeline for COVID-19 emergency planning - Supplementary Material

Joseph C. Lemaitre^[1], Kyra H. Grantz^[2], Joshua Kaminsky^[2], Hannah R. Meredith^[2], Shaun A. Truelove^[3,4], Stephen A. Lauer[2], Lindsay T. Keegan[5], Sam Shah, Josh Wills, Kathryn Kaminsky, Javier Perez-Saez[2], Justin Lessler[2], Elizabeth C. Lee[2]
^ denotes equal contribution

1 Laboratory of Ecohydrology, School of Architecture, Civil and Environmental Engineering, École Polytechnique Fédérale de Lausanne, Lausanne, Switzerland
2 Department of Epidemiology, Johns Hopkins Bloomberg School of Public Health, Baltimore, MD, USA
3 Department of International Health, Johns Hopkins Bloomberg School of Public Health, Baltimore, MD, USA
4 International Vaccine Access Center, Johns Hopkins Bloomberg School of Public Health, Baltimore, MD, USA
5 University of Utah, Department of Internal Medicine, Division of Epidemiology, Salt Lake City, Utah

Correspondence: elizabeth.c.lee@jhu.edu

Non-pharmaceutical interventions

Table S1. A suite of commonly modeled interventions, listed in order of increasing impact on transmission

Intervention	Summary	R0 reduction (%)	Reference
School Closures	Only schools are closed in this mild form of social distancing. Reduction in transmission is based on studies that estimated how influenza transmission was impacted when schools were closed due to holidays or inclement weather.	16 - 30	[1,2]
Mild Social Distancing	Similar to the Washington, D.C. response to the 1918 influenza pandemic when schools, places of worship, and places of entertainment were closed, public meetings banned, and business opening hours staggered.	21 - 33	[3]
Self Isolation and Household Quarantine	Based on the BBC Pandemic data, cases are assumed to self isolate away from the home and household members of the case self-quarantine within 2.6 - 3.6 days of case being confirmed.	26 - 37	[4]
Moderate Social Distancing	Similar to the Milwaukee, Wisconsin response to the 1918 Influenza Pandemic when all public institutions were closed - schools, places of worship, places of entertainment, and non-essential businesses.	44 - 65	[3]
Test and Isolate	Based on the rapid testing and isolation efforts put in place in South Korea in response to COVID-19.	48 - 76	[5]
Self Isolation, Household Quarantine, and Contact Tracing of acquaintances	Based on the BBC Pandemic data, cases are assumed to self isolate away from the home and household members of the case self-quarantine within 2.6 - 3.6 days of case being confirmed. It’s assumed that 90% of school, 79%	53 - 57	[4]

	of work, and 52% of other contacts are traceable.		
Self Isolation, Household Quarantine, Contact Tracing of acquaintances, and Social Distancing	Based on the BBC Pandemic data, cases are assumed to self isolate away from the home and household members of the case self-quarantine within 2.6 - 3.6 days of case being confirmed. It's assumed that 90% of school, 79% of work, and 52% of other contacts are traceable. Furthermore, mild social distancing is in place that limits contacts outside of home/school/work to at most 4 per day.	61 - 64	[4]
United Kingdom-like Lockdown	Based on the United Kingdom stay-at-home order in response to COVID-19.	71 - 83	[6]
Wuhan-like Lockdown	Based on the Wuhan stay-at-home order in response to COVID-19, where residents were not allowed to leave the city.	81 - 89	[7,8]

Health outcomes

For our work, we have been using this functionality to include age-standardized probabilities of hospitalization, ICU admission, and death; there is currently limited data on the age-specific risk of ventilation among ICU patients, and we used a single, average risk of ventilation across all locations. We reviewed the literature for relevant age-specific estimates of risk of each health outcome, and used a logistic generalized additive model (GAM) with penalized cubic spline for age and random effect for study to estimate the risk for 10-year aggregated age categories. We then apply these age-specific estimates to the population age distribution in each location to get location-specific, age-adjusted estimates. We assume that individuals of all ages are at equal risk of infection.

Table S2. Selected estimates of health outcomes risk and timings.

Event	Summary	Risk	Reference
**Hospitalization**	Probability of hospitalization given infection	2.5%, 5%, 10%	User defined; often taken to be 10x risk of death among all infected individuals
**Death**	Probability of death given infection	0.25%, 0.5%, 1%	User defined
**ICU admission**	Probability of ICU admission among those hospitalized	26.4%	[9]
		14.2%	[10]
		9.0%	[11]
**Ventilation**	Probability of invasive ventilation among those admitted to the ICU	15%	[12]
		85.8%	[10]
		88.5%	[11]

*Note that age-specific estimates of these risks and code to summarize these values can be found at https://github.com/HopkinsIDD/covidSeverity

Event	Summary	Parameters	Timing	Reference
**Hospitalization**	**Time from symptom onset to first hospitalization**	Log-mean, log-sd	1.23, 0.79	[13]
		Gamma shape, rate	0.8, 0.18	[14]
		Gamma shape, rate	0.71, 0.27	[14]
		Median, IQR	11, 8-14	[15]
		Median	5.5	[16]
**Hospital Discharge**	**Length of hospital stay** Time from hospital admission to discharge	Log-mean, log-sd	log(11.5), log(1.22)	[16]
		Median, IQR	10. 7-14	[17]
		Median, IQR	12, 10-14	[18]

		Median, IQR	12, 9-15	[15]
**Death**	**Time from symptom onset to death**	Log-mean, log-sd	2.84, 0.52	[19]
		mean, sd	22.3, 0.42	[20]
		Mean, sd	18.8, 0.45	[21]
		Median, IQR	18.5, 15 - 22	[15]
**ICU admission**	**Time from hospital admission to ICU admission** There are few direct estimates of the rate of ICU admission following hospitalization. Two small case series reported medians of 1 - 3d. Other studies estimated the time from symptom onset to ICU admission to be 10 - 12 days	Log-mean, log-sd	log(3), 0.3	[12,15,17]
**ICU discharge**	**Length of ICU stay** Time from ICU admission to ICU discharge	Log-mean, log-sd	log(8), 0.2	[15]
**Ventilation**	**Time from ICU admission to ventilation** There are few direct estimates of the rate of ventilation following ICU admission. Two studies estimated the time from symptom onset to ventilation to be 10.5 - 14.5 days, or 0 - 2 days longer than the time from symptom onset to ICU admission in each study.	Log-mean, log-sd	log(1), 0.4	[12,15]
**Extubation**	**Length of ventilation**	Log-mean, log-sd	log(7), 0.2	1 day shorter than ICU duration; one estimate of 4 patients had mean 17 days so this may be an underestimate [22]

Example YAML configuration file

Please see additional details for each of these configuration file options in the wiki page of the GitHub template repository “HopkinsIDD/COVID19_Minimal.”

```yaml
name: location-x
start_date: 2020-01-31
end_date: 2020-12-31
nsimulations: 1000
dt: 0.25
report_location_name: Location X

spatial_setup:
  base_path: data/location-x
  setup_name: location-x
  geodata: geodata.csv
  shapefile_name: shp_location_x/counties_2018.shp
  shapefile: shp_location_x/counties_2018.shp
  mobility: mobility.csv
  popnodes: pop2010
  nodenames: geoid
  census_year: 2010
  modeled_states:
    - AA
    - BB
    - CC
  include_in_report: targeted

importation:
  census_api_key: "insert_key_here"
  travel_dispersion: 3
  maximum_destinations: Inf
  dest_type : state
  dest_country : USA
  aggregate_to: airport
  cache_work: TRUE
  update_case_data: TRUE
  draw_travel_from_distribution: FALSE
  print_progress: FALSE
  travelers_threshold: 10000
  airport_cluster_distance: 80
  param_list:
```

```
    incub_mean_log: log(5.89)
    incub_sd_log: log(1.74)
    inf_period_nohosp_mean: 15
    inf_period_nohosp_sd: 5
    inf_period_hosp_mean_log: 1.23
    inf_period_hosp_sd_log: 0.79
    inf_period_hosp_shape: 0.75
    inf_period_hosp_scale: 5.367
    p_report_source: [0.05, 0.25]
    shift_incid_days: -10
    delta: 1

seeding:
 method: FolderDraw
 folder_path: importation/location-x/

interventions:
  scenarios:
    - Uncontrolled
    - SocialDistancing_fixed
    - SocialDistancing_fatigued
    - SocialDistancing_pulsed
    - SocialDistancing_checker
  settings:
    Uncontrolled:
      template: ReduceR0
      period_start_date: 2020-03-19
      period_end_date: 2020-05-14
      value:
        distribution: fixed
        value: 0
    SocialDistancing_fixed:
      template: ReduceR0
      period_start_date: 2020-03-19
      period_end_date: 2020-12-31
      value:
        distribution: uniform
        low: .71
        high: .83
    SocialDistancing_checker:
      template: ReduceR0
      affected_geoids: ["County B", "County E", "County F"]
      period_start_date: 2020-03-19
```

```
    period_end_date: 2020-12-31
    value:
      distribution: uniform
      low: .71
      high: .83
  SD_Deg1:
    template: ReduceR0
    period_start_date: 2020-03-19
    period_end_date: 2020-04-12
    value:
      distribution: uniform
      low: .71
      high: .83
  SD_Deg2:
    template: ReduceR0
    period_start_date: 2020-04-13
    period_end_date: 2020-04-26
    value:
      distribution: uniform
      low: .71*.90
      high: .83*.90
  SD_Deg3:
    template: ReduceR0
    period_start_date: 2020-04-27
    period_end_date: 2020-05-10
    value:
      distribution: uniform
      low: .71*.80
      high: .83*.80
  SD_Deg3_Road2Recovery:
    template: ReduceR0
    period_start_date: 2020-04-27
    period_end_date: 2020-05-14
    value:
      distribution: uniform
      low: .71*.80
      high: .83*.80
  SD_Deg4:
    template: ReduceR0
    period_start_date: 2020-05-11
    period_end_date: 2020-05-24
    value:
      distribution: uniform
```

```
    low: .71*.70
    high: .83*.70
SD_Deg5:
  template: ReduceR0
  period_start_date: 2020-05-25
  period_end_date: 2020-06-14
  value:
    distribution: uniform
    low: .71*.60
    high: .83*.60
SD_Deg6:
  template: ReduceR0
  period_start_date: 2020-06-15
  period_end_date: 2020-06-28
  value:
    distribution: uniform
    low: .71*.50
    high: .83*.50
SD_Deg7:
  template: ReduceR0
  period_start_date: 2020-06-29
  period_end_date: 2020-07-12
  value:
    distribution: uniform
    low: .71*.40
    high: .83*.40
SD_Deg8:
  template: ReduceR0
  period_start_date: 2020-07-13
  period_end_date: 2020-08-31
  value:
    distribution: uniform
    low: .71*.30
    high: .83*.30
SD_Deg9:
  template: ReduceR0
  period_start_date: 2020-09-01
  period_end_date: 2020-12-31
  value:
    distribution: uniform
    low: .71*.20
    high: .83*.20
SD_Pulse1:
```

```yaml
    template: ReduceR0
    period_start_date: 2020-03-19
    period_end_date: 2020-04-08
    value:
      distribution: uniform
      low: .71
      high: .83
  SD_Pulse2:
    template: ReduceR0
    period_start_date: 2020-04-30
    period_end_date: 2020-05-20
    value:
      distribution: uniform
      low: .71
      high: .83
  SD_Pulse3:
    template: ReduceR0
    period_start_date: 2020-06-11
    period_end_date: 2020-07-01
    value:
      distribution: uniform
      low: .71
      high: .83
  SD_Pulse4:
    template: ReduceR0
    period_start_date: 2020-07-23
    period_end_date: 2020-08-12
    value:
      distribution: uniform
      low: .71
      high: .83
  SocialDistancing_fatigued:
    template: Stacked
    scenarios:
      - SD_Deg1
      - SD_Deg2
      - SD_Deg3
      - SD_Deg4
      - SD_Deg5
      - SD_Deg6
      - SD_Deg7
      - SD_Deg8
      - SD_Deg9
```

```yaml
    SocialDistancing_pulsed:
      template: Stacked
      scenarios:
        - SD_Pulse1
        - SD_Pulse2
        - SD_Pulse3
        - SD_Pulse4

seir:
  parameters:
    sigma: 1 / 5.2
    gamma:
      distribution: uniform
      low: 1 / 6
      high: 1 / 2.6
    R0s:
      distribution: uniform
      low: 2
      high: 3

hospitalization:
  paths:
    output_path: hospitalization
    run_age_adjust: TRUE
  parameters:
    time_hosp: [log(7), 0.3]
    time_disch: [log(11.5), 0.20]
    time_ICU: [log(3), 0.3]
    time_ICUdur: [log(8), 0.2]
    time_ventdur: [log(7), 0.2]
    time_vent: [log(1), 0.4]
    time_onset_death: [log(17.1), 0.52]
    p_death: [.01]
    p_death_names: ["high"]
    p_hosp_inf: [0.1]

report:
  data_settings:
    pop_year: 2018
  plot_settings:
    plot_intervention: TRUE
  formatting:
    scenario_labels_short: ["UC", "SDfix", "SDfat", "SDpul", "SDchx"]
```

```
    scenario_labels:
    - "Uncontrolled"
    - "Social Distancing Fixed"
    - "Social Distancing Fatigued"
    - "Social Distancing Pulsed"
    - "Social Distancing Checker"
    scenario_colors: ["#993404", "#D95F02", "#1B9E77", "#7570B3",
"#4278cf"]
    pdeath_labels: ["1% IFR"]
    display_dates: ["2020-04-01", "2020-05-01", "2020-06-01",
"2020-07-01", "2020-08-01", "2020-09-01"]
    display_dates2: ["2020-05-01", "2020-07-01", "2020-09-01"]
```

Supplementary References:

1. Jackson ML, Hart GR, McCulloch DJ, Adler A, Brandstetter E, Fay K, et al. Effects of weather-related social distancing on city-scale transmission of respiratory viruses. medRxiv. 2020. Available: https://www.medrxiv.org/content/10.1101/2020.03.02.20027599v1.abstract

2. Cauchemez S, Valleron A-J, Boëlle P-Y, Flahault A, Ferguson NM. Estimating the impact of school closure on influenza transmission from Sentinel data. Nature. 2008;452: 750–754.

3. Bootsma MCJ, Ferguson NM. The effect of public health measures on the 1918 influenza pandemic in US cities. Proceedings of the National Academy of Sciences. 2007;104: 7588–7593.

4. Kucharski AJ, Klepac P, Conlan A, Kissler SM, Tang M, Fry H, et al. Effectiveness of isolation, testing, contact tracing and physical distancing on reducing transmission of SARS-CoV-2 in different settings. medRxiv. 2020. Available: https://www.medrxiv.org/content/10.1101/2020.04.23.20077024v1.abstract

5. Abbott S, Hellewell J, Munday JD, Chun JY, Thompson RN, Bosse NI, et al. Temporal variation in transmission during the COVID-19 outbreak. CMMID Repository. 2020. Available: https://cmmid.github.io/topics/covid19/global-time-varying-transmission.html

6. Jarvis CI, Van Zandvoort K, Gimma A, Prem K, Klepac P, Rubin GJ, et al. Quantifying the impact of physical distance measures on the transmission of COVID-19 in the UK. BMC Med. 2020;18: 1–10.

7. Zhang B, Zhou H, Zhou F. Study on SARS-COV-2 transmission and the effects of control measures in China. medRxiv. 2020. Available: https://www.medrxiv.org/content/10.1101/2020.02.16.20023770v1.abstract

8. Mizumoto K, Kagaya K, Chowell G. Early epidemiological assessment of the transmission potential and virulence of coronavirus disease 2019 (COVID-19) in Wuhan City: China, January-February, 2020. medRxiv. 2020. Available:

https://www.medrxiv.org/content/10.1101/2020.02.12.20022434v2.abstract

9. Wu C, Chen X, Cai Y, Xia J 'an, Zhou X, Xu S, et al. Risk Factors Associated With Acute Respiratory Distress Syndrome and Death in Patients With Coronavirus Disease 2019 Pneumonia in Wuhan, China. JAMA Intern Med. 2020. doi:10.1001/jamainternmed.2020.0994

10. Richardson S, Hirsch JS, Narasimhan M, Crawford JM, McGinn T, Davidson KW, et al. Presenting Characteristics, Comorbidities, and Outcomes Among 5700 Patients Hospitalized With COVID-19 in the New York City Area. JAMA. 2020. doi:10.1001/jama.2020.6775

11. Grasselli G, Zangrillo A, Zanella A, Antonelli M, Cabrini L, Castelli A, et al. Baseline Characteristics and Outcomes of 1591 Patients Infected With SARS-CoV-2 Admitted to ICUs of the Lombardy Region, Italy. JAMA. 2020. doi:10.1001/jama.2020.5394

12. Huang C, Wang Y, Li X, Ren L, Zhao J, Hu Y, et al. Clinical features of patients infected with 2019 novel coronavirus in Wuhan, China. Lancet. 2020;395: 497–506.

13. Bi Q, Wu Y, Mei S, Ye C, Zou X, Zhang Z, et al. Epidemiology and transmission of COVID-19 in 391 cases and 1286 of their close contacts in Shenzhen, China: a retrospective cohort study. Lancet Infect Dis. 2020. doi:10.1016/S1473-3099(20)30287-5

14. Zhang J, Litvinova M, Wang W, Wang Y, Deng X, Chen X, et al. Evolving epidemiology and transmission dynamics of coronavirus disease 2019 outside Hubei province, China: a descriptive and modelling study. Lancet Infect Dis. 2020. doi:10.1016/S1473-3099(20)30230-9

15. Zhou F, Yu T, Du R, Fan G, Liu Y, Liu Z, et al. Clinical course and risk factors for mortality of adult inpatients with COVID-19 in Wuhan, China: a retrospective cohort study. Lancet. 2020;395: 1054–1062.

16. Sanche S, Lin YT, Xu C, Romero-Severson E, Hengartner N, Ke R. The Novel Coronavirus, 2019-nCoV, is Highly Contagious and More Infectious Than Initially Estimated. medRxiv. 2020. doi:10.1101/2020.02.07.20021154

17. Wang D, Hu B, Hu C, Zhu F, Liu X, Zhang J, et al. Clinical Characteristics of 138 Hospitalized Patients With 2019 Novel Coronavirus-Infected Pneumonia in Wuhan, China. JAMA. 2020. doi:10.1001/jama.2020.1585

18. Guan W-J, Ni Z-Y, Hu Y, Liang W-H, Ou C-Q, He J-X, et al. Clinical Characteristics of Coronavirus Disease 2019 in China. N Engl J Med. 2020;382: 1708–1720.

19. Jung S-M, Akhmetzhanov AR, Hayashi K, Linton NM, Yang Y, Yuan B, et al. Real-Time Estimation of the Risk of Death from Novel Coronavirus (COVID-19) Infection: Inference Using Exported Cases. J Clin Med Res. 2020;9. doi:10.3390/jcm9020523

20. Dorigatti I, Okell L, Cori A, Imai N, Baguelin M, Bhatia S, et al. Report 4: severity of 2019-novel coronavirus (nCoV). Imperial College London, London. 2020. Available:

http://www.corona-portal.de/ImpColl.pdf

21. Verity R, Okell LC, Dorigatti I, Winskill P, Whittaker C, Imai N, et al. Estimates of the severity of coronavirus disease 2019: a model-based analysis. Lancet Infect Dis. 2020. doi:10.1016/S1473-3099(20)30243-7

22. Chen N, Zhou M, Dong X, Qu J, Gong F, Han Y, et al. Epidemiological and clinical characteristics of 99 cases of 2019 novel coronavirus pneumonia in Wuhan, China: a descriptive study. Lancet. 2020;395: 507–513.
